# Medical history of discordant twins and environmental etiologies of autism

**DOI:** 10.1038/tp.2016.269

**Published:** 2017-01-31

**Authors:** C Willfors, T Carlsson, B-M Anderlid, A Nordgren, E Kostrzewa, S Berggren, A Ronald, R Kuja-Halkola, K Tammimies, S Bölte

**Affiliations:** 1 Karolinska Institutet Center for Neurodevelopmental Disorders, Pediatric Neuropsychiatry Unit, Department of Women's and Children's Health, Karolinska Institutet, Solna, Sweden; 2 Center for Psychiatry Research, Stockholm County Council, Stockholm, Sweden; 3 Prima Child and Adult Psychiatry, Stockholm, Sweden; 4 Department of Molecular Medicine and Surgery, Center of Molecular Medicine, Karolinska Institutet, Solna, Sweden; 5 Department of Clinical Genetics, Karolinska University Hospital, Solna, Sweden; 6 Child and Adolescent Psychiatry, Stockholm County Council, Stockholm, Sweden; 7 Genes Environment Lifespan laboratory, Department of Psychological Sciences, Birkbeck, University of London, London, UK; 8 Department of Medical Epidemiology and Biostatistics, Karolinska Institutet, Solna, Sweden

## Abstract

The environmental contributions to autism spectrum disorder (ASD) and their informative content for diagnosing the condition are still largely unknown. The objective of this study was to investigate associations between early medical events and ASD, as well as autistic traits, in twins, to test the hypothesis of a cumulative environmental effect on ASD risk. A total of 80 monozygotic (MZ) twin pairs (including a rare sample of 13 twin pairs discordant for clinical ASD) and 46 dizygotic (DZ) twin pairs with varying autistic traits, were examined for intra-pair differences in early medical events (for example, obstetric and neonatal factors, first year infections). First, differences in early medical events were investigated using multisource medical records in pairs qualitatively discordant for ASD. The significant intra-pair differences identified were then tested in relation to autistic traits in the remaining sample of 100 pairs, applying generalized estimating equations analyses. Significant association of the intra-pair differences in the MZ pairs were found for the cumulative load of early medical events and clinical ASD (*Z*=−2.85, *P*=0.004) and autistic traits (*β*=78.18, *P*=0.002), as well as infant dysregulation (feeding, sleeping abnormalities, excessive crying and worriedness), when controlling for intelligence quotient and attention deficit hyperactivity disorder comorbidity. The cumulative load of early medical events in general, and infant dysregulation in particular, may index children at risk of ASD owing to non-shared environmental contributions. In clinical practice, these findings may facilitate screening and early detection of ASD.

## Introduction

Autism spectrum disorder (ASD) is an increasingly diagnosed neurodevelopmental condition with a heterogeneous and life-long presentation. It is exclusively behaviorally defined by an early onset of persistent difficulties in social communication and interaction, alongside repetitive, restricted interests and activities causing impairment in daily life.^1^ Traits of autism are continuously distributed in the general population and overlap with clinical phenotypes of ASD.^2, 3, 4^ Comorbidity with other neurodevelopmental disorders is common in ASD.^5^ Symptoms of ASD are typically measurable at the age of 12 months, still most cases are diagnosed much later.^6^ Early behavioral signs of ASD are limited in sensitivity and specificity, and current screening tools show low positive predictive values.^7^ Establishing reliable early detection of the condition remains a clinical research priority, as early identification typically ensures access to intervention and facilitates improved outcomes.^8^

Twin and family studies have shown both genetic and environmental factors to be influential in ASD etiology with heritability estimates ranging from 38 to 55%^9,10^ up to 95%,^3^ and non-shared environmental (NSE) factors to be the predominant environmental influence.^9, 11^ For autistic traits, a British population-based twin study reported evidence that twins with more neonatal problems had more autistic traits. For example, lower birth weight was associated with more autistic traits in the social domain in middle childhood.^12^ Multiple single environmental risks have been implicated in ASD, both shared environment (SE) factors, such as maternal valproate intake, exposure to toxic chemicals, enhanced steroidogenic activity, gestational diabetes and maternal obesity, and NSE factors, such as birth injury and trauma, and neonatal anemia.^13, 14, 15, 16^ Bolton *et al.*^17^ in their study on the relevance of obstetric complications on ASD etiology, found that rather than playing any primary causal role, the birth complications associated with autism either represent an epiphenomenon of the condition or derive from shared risk factor(s). A number of environmental risk factors have been suggested to lead to epigenetic dysregulation, and increase ASD risk, such as prenatal exposure to valproate.^18^ Overall, the current evidence favors a multiple risk factor threshold model hypothesizing that an increased exposure to a composition of environmental stressors increases the risk for ASD in individuals with a genetic vulnerability.^19^ Further, it is suggested that the cumulative load of risk factors correlates with the severity of ASD.^19^

As genetic factors influence how individuals respond to different environmental exposures, limited control over the individual genetic factors has been a major weakness of most studies investigating environmental risks for ASD. As monozygotic (MZ) twins are identical on a DNA sequence level, with the exception of putative post-twinning *de novo* mutations, all differences in phenotypes seen in MZ twin pairs are attributable to effects of NSE.^20^ Particularly studies investigating MZ pairs discordant for the phenotype in question are powerful to elucidate environmental risk factors.^21^ Only a few studies have applied a discordant MZ twin pair design in ASD as reviewed by Ronald and Hoekstra.^11^

The objective of this study was to explore associations between potential environmental risk factors, ASD and autistic traits. Specifically, we sought to identify early medical events likely to be caused by environmental factors that are associated with ASD and autistic traits, and test the hypothesis of their cumulative effect on risk status. For this purpose, we scrutinized the early medical histories of a rare and informative sample of 13 MZ twin pairs discordant for clinical ASD. The findings were then cross-validated in a separate larger cohort of MZ and dizygotic (DZ) twins discordant for autistic traits (*N*=100 pairs), operationalizing them in a cumulative multifactorial model. The results of this study provide information about early medical events and adverse behavioral manifestations that may index children at risk for ASD, owing to NSE influences. Such evidence could be used to facilitate early detection of ASD, as it might either further support or discourage an existing diagnostic suspicion.

## Materials and methods

### Diagnostic and behavioral assessments

The participants were diagnosed by three experienced clinicians according to DSM-5 criteria using clinical consensus supported by results from the Autism Diagnostic Interview – Revised,^22^ the Autism Diagnostic Observation Schedule Second Edition,^23^ the Kiddie Schedule for Affective Disorders and Schizophrenia^24^ or the Diagnostic Interview for attention deficit hyperactivity disorder (ADHD) in Adults.^25^ The Wechsler Intelligence Scales for Children or Adults, Fourth Editions,^26, 27^ or the Leiter-revised scales^28^ in combination with the Peabody Picture Vocabulary Test Third Edition^29^ (in cases of low verbal abilities) and the parent-rated Adaptive Behavior Assessment Scale, 2nd Edition,^30^ were applied to assess general cognitive and verbal abilities and adaptive functional level. Autistic traits were measured by the parent report version of the Social Responsiveness Scale-2 (SRS-2) using total raw scores as recommended for research settings.^31^ The SRS-2 includes 65 Likert scaled items, generating a total maximum score of 195. The questions in SRS-2 focus on the individual's behavior during the past 6 months and inquiries about autistic traits, such as alterations in social cognition, social awareness, social motivation, social communication and inflexible, repetitive behavior. Increasing scores on the SRS-2 indicate more autistic traits. The SRS-2 has demonstrated satisfactory-to-excellent psychometric properties for test–retest reliability (0.80–0.97), interrater reliability (0.75–0.95) and convergent validity (0.35–0.58).^32, 33^

The exposure of early medical events were assessed in relation to either quantitative or qualitative discordance for ASD. Qualitative discordance was defined as only one twin within a pair meeting the diagnostic criteria of ASD, and quantitative discordance as at least one point intra-pair difference on the SRS-2 total score. In the first step of analyses, intra-pair medical event differences in a sample of qualitative ASD discordant twins was examined and compared with TD controls. In the second step of analyses, the association between identified medical events (exposure) and quantitative discordance for ASD (outcome) was assessed.

### Participants

The study was approved by the National Swedish Ethical Review Board and all the participants and/or their legal guardians gave written informed consent. A total of 126 twin pairs (80 MZ, 46 DZ) were recruited from the ‘Roots of Autism and ADHD Twin Study in Sweden' described elsewhere in detail.^34^

#### ASD discordant MZ twin sample

The first analysis step (exploratory) included 13 MZ twin pairs discordant for clinical ASD and 13 MZ typically developing (TD) control pairs (*n*=52) matched for sex (16 males and 10 females in each group). Among the twins with ASD, five had a comorbid diagnoses of ADHD. The total intelligence quotient (IQ) in the ASD discordant twin sample ranged from 40 to 121 (*M*=89.2), and 81 to 123 (*M*=98.1) in the TD control twins. A quantitative intra-pair difference was found for all but two of the behavioral measures, with the largest effect size for the SRS-2 total score (*r*=0.85, *Z*=−3.06, *P*=0.002), that was higher in the ASD twin, and the second largest for full-scale IQ (*r*=0.80, *Z*=−2.87, *P*=0.004), that was higher in the non-ASD twin. Effect sizes were calculated according to *r*=*Z*/√*N*. These intra-pair differences were not found in the 13 TD MZ pairs (Supplementary Tables 1 and 2).

#### Autistic trait discordant MZ and DZ twin sample

In the second step of analysis (confirmatory), we included 100 twin pairs quantitatively discordant for autistic traits, 54 MZ pairs and 46 DZ pairs. In the MZ group, 29 participants were assessed positive for ASD, 32 positive for ADHD and 13 were positive for both disorders. The equivalent numbers in the DZ groups were 23 participants with ASD, 35 with ADHD and 13 with ASD and ADHD combined. IQ ranged from 42 to 142 in the total sample, with a mean of 96.6 in the MZ group and a mean of 97.7 in the DZ group. The percentage of females was 44% in the MZ group and 46% in the DZ group (Table 1).

### Zygosity

Zygosity was determined by genotyping of saliva or whole-blood derived DNA using Infinium Human-CoreExome chip (Illumina, San Diego, CA, USA). The estimating identity by descent was analyzed using the PLINK software^35^ after quality control and removal of SNPs with minor allele frequency less than 0.05 within the samples. All pairs of DNA samples showing π ⩾0.99 were considered as monozygotic pairs. For few pairs, a short tandem repeat kit (Promega Powerplex 21) or zygosity questionnaire (six pairs) was used.

### Medical and developmental history

For the exploratory first step of analyses comparing qualitative ASD discordant MZ twins to TD MZ twin controls, detailed information on medical and developmental history with focus on the first 5 years of life was collected from questionnaires and medical records. Intraclass Correlation Coefficient (ICC) analysis^36^ showed good agreement between the questionnaire and medical record information. The agreement was moderate for cumulative load of early medical events (ICC=0.55, 95% confidence interval=0.22–0.75), substantial for dysregulation (ICC=0.76, 95% confidence interval=0.58–0.86) and almost perfect for birth weight (ICC=0.93, 95% confidence interval=0.88–0.96). Medical records comprised prenatal records, birth records, records of pediatric clinics and medical and psychiatric care unit documentation. Medical history in the total sample was assessed from a parent reported questionnaire. The 114 items of the questionnaire covered medical history events such as current diagnosis and medications, family situation at birth, family medical history, pre-, peri- and postnatal factors, child disease history and diagnostic tests. Most questions were yes/no questions with a request to specify for positive answers. In some cases, the questions on paper were followed up with an interview with clinical geneticists for clarifications. The questionnaire data and health care records for the subsample were reviewed independently by four researchers (TC, B-MA, AN, CW) with different clinical backgrounds (that is, child and adolescent psychiatrist, neuropediatrician, clinical geneticist and psychologist). Two of the researchers (B-MA, AN) were blinded for clinical diagnoses, hypotheses and planned analysis. Intra-pair differences for the frequency and age of onset for developmental alterations, medical complications and life events were noted. In the case of contradictory information, a decision-making hierarchy was applied on the basis of the presumed validity of the information sources. For example, birth weight data from the birth record was considered more valid than the same information from the questionnaire. Registered medical history events were coded binary (‘1' for present, ‘0' for not present in each individual; Supplementary Table 3). In addition, the medical history events were categorized according to the type of events (for example, delivery-related factors and minor and frequent infections) and summarized into ordinal data. All medical history events, that by the four researchers, were identified as differing within ASD discordant pairs (that is, present in only one twin in a pair), were added up to generate a cumulative load of early medical events for each individual (31 factors). To deal with missing data (NA), the sum of individual factors was divided with the total number of variables, subtracted with the individual number of missing variables according to the formula below.


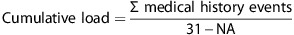


The mean number of missing data in the sample was 1.7 (range 0–23). In addition to the binary code of intra-pair birth weight differences, the quantitative information (differences in grams) was used in a separate analysis of birth weight.

### Statistical analysis

Statistics were computed using IBM SPSS 22 and the drgee package^37^ in R version 3.2.4. Owing to the sample size, nonparametric tests were used (that is, Wilcoxon signed-rank test for continuous and ordinal data and McNemar's test for binary data) to assess the within-pair effect for the identified medical events in the qualitative discordant MZ subsample. All the tests in the first step of analyses were two-tailed. In the second step of analyses of the quantitative discordant sample, a conditional linear regression model, based on generalized estimating equations analysis^38^ was fitted. The conditional model estimates the within-pair effect, adjusting for all shared factors in twin pairs (for example, all genetic factors in MZ pairs).^39^ One-tailed tests were applied for the regression analyses. Autistic trait severity (SRS-2 total score) was used as outcome variable in the model. The model was adjusted for the following covariates: full-scale IQ, ADHD diagnoses (binary) and sex. In addition, the sample was split into zygosity groups comparing the within-pair effects in the MZ and DZ pairs.

## Results

### Early medical history in twins discordant for clinical ASD

**Figure 1:**
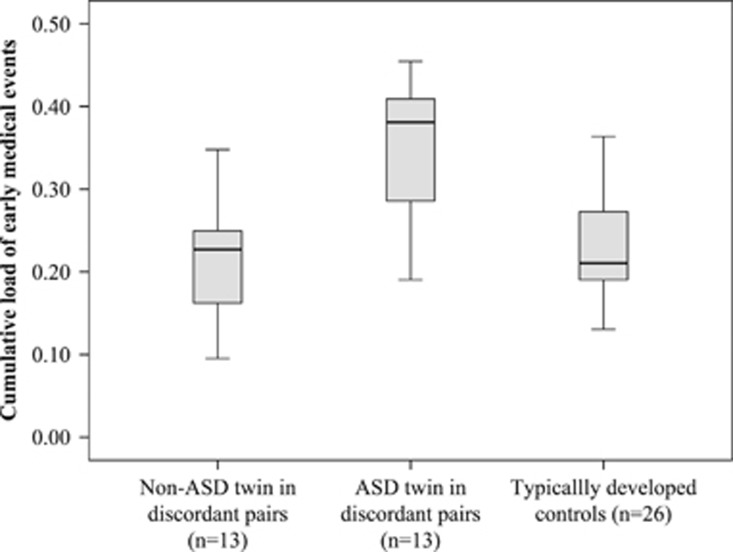
The adjusted cumulative load of medical events in ASD discordant and typically developed MZ twins. ASD, autism spectrum disorder; MZ, monozygotic.

Single early medical factors, likely to be caused by NSE, that discriminated between twins in qualitative ASD discordant pairs were dysregulation during the first year of life (comprising feeding and sleeping problems, excessive crying and worrying; *Z*=−2.56, *P*=0.011) and birth weight (*Z*=−2.20, *P*=0.028) (Table 2). MZ twins with ASD and their non-ASD co-twins also differed for the cumulative load of early medical events (*Z*=−2.85, *P*=0.004; Table 2, Figure 1). None of these intra-pair differences were observed in the 13 TD MZ pairs (Figure 1).

### Early medical history in autistic traits discordant twins

**Figure 2:**
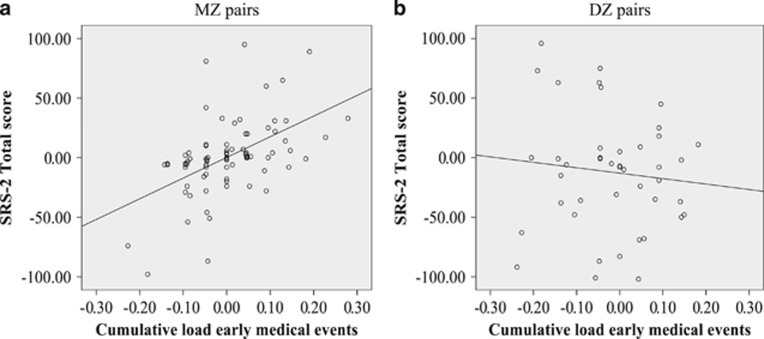
The association between intra-pair differences in SRS-2 total score and cumulative load of early medical events in MZ and DZ pairs. The graphs show a significant positive correlation (Pearsons's *R*^2^) for MZ pairs (**a**) and a nonsignificant correlation for DZ pairs (**b**). DZ, dizygotic; MZ, monozygotic; SRS-2, Social Responsiveness Scale—2nd edition.

A single early medical factor, likely to be caused by NSE, that correlated with the extent of autistic traits was dysregulation during the first year of life (*β*=31.75, *P*=0.03). The association for birth weight and ASD traits was not significant, but showed a similar trend (*β*=−0.01, *P*=0.05; See Table 3). There was an association between the intra-pair differences in cumulative load of early medical events and autistic traits in the MZ pairs (*β*=78.18, *P*=0.002; Table 3). This effect indicates a three point intra-pair increase for autistic traits on the SRS-2 for every single medical event's difference. All models were adjusted for full-scale IQ and ADHD diagnoses, with no significant gender effect found. The association between autistic traits and the cumulative load of early medical events hold also when excluding the dysregulation variable from the cumulative load score (*β*=54.42, *P*=0.005). No significant associations were seen in the DZ pairs (Table 3; Figure 2).

## Discussion

In this study, we show that early medical events are associated with clinical ASD phenotypes and continuously distributed autistic traits. The latter was particularly true for early dysregulation and the cumulative load of a variety of early adverse medical events. Using co-twin modeling, we were able to control for genetic and SE factors, endorsing that the found associations were indeed driven by NSE factors within the MZ twin pairs. Our study show the novel finding of a cumulative environmental effect on ASD risk, when controlling for genetics. In addition, we were able to replicate earlier findings of early dysregulation as a precursor of behavioral problems and autistic traits. A previous large population-based study including over 4000 infants reported that early regulatory problems predict behavioral problems (that is, external, internal and attentional problems) later in life,^40^ and a Swedish clinical study found that regulatory issues were frequent in children later diagnosed with ASD.^41^ Our results advance these results because they demonstrate that an association between early regulation difficulties and ASD exists independent of genetic influences. Earlier studies were unable to differentiate genetic and environmental effects on these phenomena. In line with a previous community twin study,^12^ we found that an increased total load of early medical events is associated with autistic traits. Nevertheless, the associations identified in the current studies were substantially stronger.

Our study partly supports previous results indicating low birth weight to be associated with ASD risk.^20^ We found a trend for intrauterine growth to be associated with clinical ASD and autistic traits. However, although earlier studies deemed low birth weight a consequence of genetic factors, our data indicate environmental factors as cause. In addition to highlighting NSE factors causing early developmental adversity in ASD, our results also suggest early adversity factors being of potential relevance as early red flags for autism risk evaluation. Although symptoms of ASD emerge as early as 12 months of age,^6^ and ASD can quite reliably be diagnosed between 24 and 36 months of age, many children are not diagnosed until the age of 5 or 6 years. This is unfortunate as early detection is a prerequisite for early intervention,^42^ which is associated with better outcomes.^43^ The screening tools available for ASD before the age of 24 months are limited in sensitivity and specificity,^43^ and as the condition is exclusively behaviorally defined, the tools do not include early medical features. Our data indicate that taking into account the cumulative load of early medical factors might strengthen or discourage a suspicion of ASD, at least in a minority of cases. However, further research should explore how these general risk factors could be practically useful for identifying a specific condition such as ASD.

Our study harbors some noteworthy strengths. For instance, although there is a large phenotypic as well as etiological overlap between ASD, intellectual disability and ADHD,^44^ few ASD studies have taken comorbid diagnoses into consideration, either excluding comorbidity, not assessing it at all or limiting the ASD sample to those individuals without intellectual disabilities. This study controlled for the effects of IQ as well as diagnosis of ADHD, indicating that results are specific to ASD. Interestingly, findings were not limited to clinical ASD phenotypes, but equally valid for autistic traits, providing further support for the notion that ASD forms the extreme end of a trait continuum.^2, 3, 4^ The importance of investigating mental health from a quantitative perspective is endorsed by the American National Institutes of Health Research Domain Criteria approach, attempting to classify mental disorders incorporating multiple dimensional outcomes rather than single categorical diagnoses. A growing body of literature supports that several currently exclusively behavioral defined psychiatric diagnoses could be enriched and clarified by multidimensional information, and a medical and environmental factor association specifier has been added to the ASD diagnostic criteria in the fifth edition of DSM.^45, 46^ In this regard, our findings endorse that information on the cumulative load of early medical events might qualify to be specified in the context of ASD assessment.

This study has several limitations that need to be addressed. First, the cumulative load of early medical events, as well as dysregulation, might not be primary or directly acting environmental risk factors for ASD, such as maternal valproate intake. These factors might be secondary (or tertiary), although they precede an ASD diagnosis, as we in this study are unable to draw firm conclusions about the possible primary factors. Second, we cannot finally exclude that possible selection and confirmation bias, and reverse causation limit the generalizability of our conclusions. However, as we use medical records as a source of information, we assume to have collected reliable data of a child's medical history, hence eliminating recall bias. Two of the abstractors of the medical records were unblinded to the diagnosis of the twins. Although, there were no systematic differences between the data abstracted by the blinded vs the non-blinded clinicians, and such a bias is unlikely for the autistic traits analyses, some degree of confirmation bias cannot be ruled out. Third, although we stress here the significance of NSE, as we apply a high genetic control, rare post-twining *de novo* mutations are acknowledged and may contribute to a minority of cases of discordance. Fourth, this study is unable to weight the different factors summing up to the cumulative load, making our approach crude. Fifth, one might consider the sample size of the ASD discordant pairs included in the first step of the study to be small. However, MZ twin designs offer a high degree of control for confounders, and our ASD discordant sample actually represents a rather large minority of those discordant pairs in Sweden.^47^ Sixth, despite the advantages of twin studies, twins might not always be representative of the general population, making cross-validation of twin findings in singletons desirable for confirm generalizability. For example, a factor such as shared or non-shared placenta, could have a MZ twin-specific NSE effect that we have not been able to control for in this study. Nevertheless, twinness in itself has not been shown to be a risk factor for ASD,^48^ nor autistic traits.^49^

In summary, we found (i) that the load of adverse early medical events is associated with autistic traits and ASD; (ii) a link between autistic traits, ASD and early dysregulation; (iii) that these early medical events are likely to be driven by NSE; and (iv) that the cumulative load of early medical events could be informative in the early detection of ASD. Our findings require an independent follow-up in epidemiological samples, for confirmation of the results and to investigate the specificity with regard to other neurodevelopmental disorders.

## Figures and Tables

**Table 1:** Sample characteristics

*Samples*	N *pairs*	*Age mean (range)*	*Sex:* n *subjects*	*ASD cases:* n *subjects*	*IQ full-scale mean (range)*
MZ discordant for ASD diagnoses	13	14.4 y (9–20)	Male: 16 Female: 10	ASD cases: 13 ADHD cases: 6 Cases with comorbid ASD and ADHD: 5	89.2 (40–121)
					
MZ TD	13	15.8 y (10–18)	Male: 16 Female: 10	ASD cases: — ADHD cases: —	98.1 (81–123)
					
MZ quantitative discordant	54	14.9 y (8–28)	Male: 60 Female: 48	ASD cases: 29 ADHD cases: 32 Cases with comorbid ASD and ADHD: 13	96.6 (58–142)
					
DZ quantitative discordant	46	14.3 y (8–25)	Male: 49 Female: 43	ASD cases: 23 ADHD cases: 35 Cases with comorbid ASD and ADHD: 13	97.7 (42–138)

Abbreviations: ADHD, attention deficit hyperactivity disorder; ASD, autism spectrum disorder; DZ, dizygotic; IQ, intelligence quotient; MZ, monozygotic; TD, typically developed; y, year.

**Table 2:** List of the non-shared variables and categories included in the cumulative load of early medical events and intra-pair differences in MZ pairs qualitative discordant for ASD (*n*=13 pairs)

*Categories*	*Wilcoxon sign-rank test*	*Variables*	*McNemar's/Wilcoxon sign-rank test*
Delivery related factors	*Z*=−1.34 *P*=0.180	Apgar 5 min	—
		Fetal distress	—
		Breech birth	*P*=0.625
			
Minor medical neonatal factors	*Z*=−1.51 *P*=0.131	Hypoglycemia	—
		Hyperbilirubinemia	*P*=1.00
		Oxygen treatment	—
		Iron depletion	—
		Thrombocytopenia	—
			
Growth at birth	—	Birth weight	*Z*=−2.20 *P*=0.028*
			
Microcephaly	—	Head circumference relative to length	*P*=0.219
			
Minor and frequent infections	*Z*=−0.58 *P*=0.564	Frequent ear infections	*P*=1.00
		Infections asthma before 5 y	*P*=1.00
		Gastroenteritis <2 y	—
			
Serious infections <2 y	*Z*=0.00 *P*=1.00	Pyelonephritis <2 y	—
		Septicemia <2 y	*P*=1.00
			
Total allergy	—	Eczema <5 y	*P*=1.00
		Allergy <5 y	—
			
Total epilepsy <5 y	*Z*=−1.41 *P*=0.157	Epilepsy <5 y	—
		Seizures first year	*P*=0.50
			
Serious medical conditions first year	*Z*=−1.00 *P*=0.317	Cerebral hemorrhage	*P*=1.00
		Cerebral paresis	—
		Hydrocephalus	—
			
Congenital heart and vessel malformations	—	Heart and large vessels malformation, Cerebral AVM	—
Brain atrophy	—	Brain atrophy	—
Head contusion	—	Head contusion <3 y	—
Visual impairments	—	Glasses	*P*=1.00
			
Dysregulation <1 y	*Z*=−2.56 *P*=0.011*	Poor sleep	*P*=0.063
		Feeding disabilities	*P*=0.125
		Frequent vomiting	—
		Crying a lot	*P*=0.250
		Worried <1 y	—
			
The cumulative load of early medical events	*Z*=−2.85 *P*=0.004**	Including all above-listed variables	

Abbreviations: ASD, autism spectrum disorder; AVM, arteriovenous malformation; MZ, monozygotic; y, year.

*P<0.05, **P<0.01.

**Table 3:** Associations between SRS total score and cumulative load of medical events, dysregulation problems and birth weight, including IQ and ADHD diagnosis as covariates

*Variables*		*MZ (*n*=54 pairs)*			*DZ (*n*=46 pairs)*		
		β	*s.e.*	P	β	*s.e.*	P
Exposure variable:	Cumulative load	78.18	26.59	0.002**	−8.71	46.03	0.43
Covariate 1:	IQ	−0.19	0.25	0.22	−1.20	0.29	<0.001***
Covariate 2:	ADHD	3.30	5.64	0.26	39.18	8.09	<0.001***
							
Exposure variable:	Dysregulation	31.75	16.2	0.03*	11.85	20.60	0.28
Covariate 1:	IQ	−0.33	0.24	0.08	−1.14	0.30	<0.001***
Covariate 2:	ADHD	2.78	5.87	0.32	40.63	8.22	<0.001***
							
Exposure variable:	Birth weight	−0.01	0.01	0.05	−0.01	0.01	0.30
Covariate 1:	IQ	−0.39	0.24	0.05	−1.19	0.28	<0.001***
Covariate 2:	ADHD	0.71	5.05	0.44	40.10	7.90	<0.001***

Abbreviations: ADHD, attention deficit hyperactivity disorder; *β**, **β* estimates; DZ, dizygotic; IQ, intelligence quotient; MZ, monozygotic; SRS-2, Social Responsiveness Scale—2nd edition; s.e., standard error.

*P<0.05, **P<0.01, ***P<0.001.
