# Supplementary material for: Medical history of discordant twins and environmental etiologies of autism
Source: Transl Psychiatry. 2017 Jan 31;7(1):e1014–. doi: 10.1038/tp.2016.269 (PMC5299390; doi:10.1038/tp.2016.269)
Supplement: Supplementary Material [file tp2016269x1.doc]

Supplemental Material

**Table 1.** Intra-pair differences for MZ pairs discordant for ASD (n=13).

| **Outcome measures**  **(Instrument; Sub-scale)** | **ASD twin** **MD (IQR)** | **Non-ASD twin** **MD (IQR)** | **Wilcoxon Ranked sign test** | **Effect size (r=Z/√N)** |
| --- | --- | --- | --- | --- |
| ADOS-2; Social affect | 8 (4.5-10.5) | 2 (1-5) | Z=-2.37, *p=* .018* | .66 |
| ADOS-2; Restricted and repetitive behavior | 1 (1-2.5) | 0 (0-2) | Z=-2.5. *p=* .013* | .69 |
| ADOS-2; Comparison score | 5 (2.5-7) | 1 (1-2.5) | Z=-2.71, *p=* .007** | .75 |
| ADI-R; Reciprocal social interaction | 12 (6.5-19) | 5 (0.5-11.5) | Z=-2.3. *p* =.021* | .64 |
| ADI-R; Communication | 9 (6-14.5) | 6 (2.5-7.5) | Z=-2.2. *p* =.028* | .61 |
| ADI-R; Restricted, repetitive, and stereotyped behavior | 3 (1.5-4.5) | 0 (0-1) | Z=-2.41,  *p* =.016* | .67 |
| ADI-R; Age of first manifestation | 2 (0.5-4.5) | 2 (0-3.5) | Z=-1.79, *p=* .074 | .50 |
| SRS-2; Total score | 86 (67-109) | 31 (22-41) | Z=-3.06, *p* =.002** | .85 |
| ABAS-II; Conceptual ability | 59 (50-66) | 82 (73-93) | Z=-2.76,  *p=* .006** | .77 |
| ABAS-II; Social ability | 52 (50-82) | 83 (68-86) | Z=-2.05,  *p=* .041* | .57 |
| ABAS-II; Practical ability | 70 (53-78) | 88 (70-102) | Z=-2.48, *p*=.013* | .69 |
| ABAS-II; General ability index | 61 (48-75) | 82 (72-93) | Z=-2.55, *p*=.011* | .71 |
| Full scale IQ | 76 (70-97) | 102 (82-113) | Z= -2.87, *p*=.004** | .80 |
| Verbal IQ | 87 (71-105) | 95 (88-105) | Z=-1.89, *p*=.059 | .52 |
| Non-verbal IQ | 84 (72-95) | 100 (80-116) | Z=-2.45, *p*=.014* | .68 |

Note: MD=Median; IQR=Interquartile Range; ADOS-2=Autism Diagnostic Observation Scale - 2^nd^ edition; ADI-R=Autism Diagnostic Interview – Revised; ASD=Autism Spectrum Disorder; IQ= Intelligence Quotient; SRS-2=Social Responsiveness Scale - 2^nd^ edition; ABAS-II=Adaptive Behavior Assessment System - 2^nd^ edition

**Table 2**. Table with intra-pair differences for MZ control pairs (n=13 pairs).

| **Outcome measures** | **Twin 1** **MD (IQR)** | **Twin 2** **MD (IQR)** | **Wilcoxon signed**  **rank test (n=13)** | **Effect size (r=Z/√n)** |
| --- | --- | --- | --- | --- |
| SRS-2 total score | 16 (11-31) | 13 (8-24) | Z=-.76; *p*=.44 | 0.21 |
| ABAS-II Conceptual ability | 92 (85-112) | 106 (81-116) | Z=-.63; *p*=.52 | 0.17 |
| ABAS-II Social ability | 97 (89-103) | 96 (81-111) | Z=-1.68; *p*=.92 | 0.47 |
| ABAS-II Practical ability | 100 (94-116) | 108 (90-119) | Z=-.31; *p*=.75 | 0.09 |
| ABAS-II General ability index | 96 (92-112) | 106 (88-116) | Z=-.42; *p*=.67 | 0.13 |
| Full scale IQ | 96 (88-104) | 95 (89-108) | Z=-.175; *p*=.86 | 0.05 |
| Verbal IQ | 96 (88-100) | 94 (90-103) | Z=-.76; *p*=.45 | 0.23 |
| Non-verbal IQ | 104 (87-108) | 97 (90-111) | Z=-.46 ; *p*=.64 | 0.13 |

Note: MD=Median; IQR=Interquartile Range; SRS-2=Social Responsiveness Scale 2^nd^ edition; ABAS-II=Adaptive Behavior Assessment System, 2^nd^ edition; IQ= Intelligence Quotient

**Table 3.** List of the non-shared variables and categories included in “the cumulative load of early medical events”, including a column describing how the equivalent questions from the questionnaire were selected.

| **Category** | **Wilcoxon, signed rank test** | **Variables included** | **McNemar/** **Wilcoxon signed rank test** | **Defined in total load of NSE factors based on questionnaire data** |
| --- | --- | --- | --- | --- |
| Delivery related factors | Z=-1.34  *p*=.180 | Apgar 5 min | - | Apgar <7 = 1 |
|  |  | Fetal distress | - | Reported positive = 1 |
|  |  | breech birth | *p=*.625 | Reported positive = 1 |
| Minor medical neonatal factors | Z=-1.51  *p*=.131 | Hypoglycemia | - | Reported positive = 1 |
|  |  | Hyperbilirubinemia | *p*=1.00 | Reported positive = 1 |
|  |  | Oxygen treatment | - | Reported positive = 1 |
|  |  | Iron depletion | - | Reported positive = 1 |
|  |  | Thrombocytopenia | - | Reported positive = 1 |
| Dysregulation 1st y | Z=-2.56  *p*=.011* | Poor sleep | *p=*.063 | Reported positive = 1 |
|  |  | Feeding disabilities | *p=*.125 | Reported positive = 1 |
|  |  | Frequent vomiting | - | Reported positive = 1 |
|  |  | Crying a lot | *p*=.250 | Reported positive = 1 |
|  |  | Worried 1st y | - | Reported positive = 1 |
| Dysregulation >5y | - | Bad appetite >5y | - | Excluded |
| Growth at birth | - | Birth weight | Z=-2.20  *p=*.028* | Lower birth weight in comparison to co-twin  (≥10 gr) = 1 |
| Microcephaly | - | Head circumference relative to length (smaller) | *p=*.219 | Smaller than co-twin = 1 |
| Sensory and motoric development | Z=-1.63  *p*=.102 | Delayed motor development >5y | *p=*.250 | Excluded |
|  |  | Boel test 8 w | - | Excluded |
| Cognitive and/or behavioral impairments | Z=-2.00  *p*=.046* | Need of assistance kindergarten | - | Excluded |
|  |  | Delayed cognitive development as assessed at school start | *p* .125 | Excluded |
|  |  | Hyperactivity/restlessness | *p* 1.00 | Excluded |
| Minor and frequent infections | Z=-.58  *p*=.564 | Frequent ear infections | *p* 1.00 | Reported positive = 0 (inverted) |
|  |  | Infections asthma before 5y | *p* 1.00 | Reported positive = 1 |
|  |  | Gastroenterit <2y | - | Reported positive = 1 |
| Serious infections <2y | Z=.00  *p*=1.00 | Pyelonephritis <2y | - | Reported positive = 1 |
|  |  | Septicemi <2y | *p* 1.00 | Reported positive = 1 |
|  |  | Eczema <5y | *p* 1.00 | Reported positive = 1 |
|  |  | Allergy <5y | - | Reported positive = 0 (inverted) |
| Total epilepsy >5y | Z0-1.41  *p*=.157 | Epilepsy <5y | - | Reported positive = 1 |
|  |  | Seizures 1st y | *p* .50 | Reported positive = 1 |
| Serious medical conditions 1st y | Z -1.00  *p*=.317 | Cerebral hemorrhage | *p* 1.00 | Reported positive = 1 |
|  |  | Cerebral paresis | - | Reported positive = 1 |
|  |  | Hydrocephalus | - | Reported positive = 1 |
| Congenital heart and vessel malformations | - | Heart and large vessels malformation, Cerebral AVM | - | Reported positive = 1 |
| Brain atrophy | - | Brain atrophy | - | Reported positive = 1 |
| Head contusion | - | Head contusion <3y | - | Reported positive = 1 |
| Visual impairments | - | Glasses | *p* 1.00 | Reported positive = 1 |
| The cumulative load of early medical events | Z=-2.85  *p=*.004** | Including all above listed variables | | |

Note: y= year
